# The cytotoxic effects of regorafenib in combination with protein kinase D inhibition in human colorectal cancer cells

**DOI:** 10.18632/oncotarget.2938

**Published:** 2014-12-03

**Authors:** Ning Wei, Edward Chu, Shao-yu Wu, Peter Wipf, John C. Schmitz

**Affiliations:** ^1^ Division of Hematology-Oncology, Department of Medicine, University of Pittsburgh School of Medicine, Pittsburgh, PA, USA; ^2^ Cancer Therapeutics Program, University of Pittsburgh Cancer Institute, University of Pittsburgh, Pittsburgh, PA, USA; ^3^ Department of Chemistry, University of Pittsburgh, Pittsburgh, PA, USA

**Keywords:** protein kinase D, regorafenib, human colorectal cancer, apoptosis, NF-κB

## Abstract

Metastatic colorectal cancer (mCRC) remains a major public health problem, and diagnosis of metastatic disease is usually associated with poor prognosis. The multi-kinase inhibitor regorafenib was approved in 2013 in the U.S. for the treatment of mCRC patients who progressed after standard therapies. However, the clinical efficacy of regorafenib is quite limited. One potential strategy to improve mCRC therapy is to combine agents that target key cellular signaling pathways, which may lead to synergistic enhancement of antitumor efficacy and overcome cellular drug resistance. Protein kinase D (PKD), a family of serine/threonine kinases, mediates key signaling pathways implicated in multiple cellular processes. Herein, we evaluated the combination of regorafenib with a PKD inhibitor in several human CRC cells. Using the Chou-Talalay model, the combination index values for this combination treatment demonstrated synergistic effects on inhibition of cell proliferation and clonal formation. This drug combination resulted in induction of apoptosis as determined by flow cytometry, increased PARP cleavage, and decreased activation of the anti-apoptotic protein HSP27. This combination also yielded enhanced inhibition of ERK, AKT, and NF-κB signaling. Taken together, PKD inhibition in combination with regorafenib appears to be a promising strategy for the treatment of mCRC.

## INTRODUCTION

Colorectal cancer (CRC) is the second leading cause of cancer mortality in the U.S., and in 2014, it is expected that about 45,000 deaths will be attributed to this disease [1]. Approximately 25% of CRC patients will have metastatic disease at the time of diagnosis, and an additional 40-50% of patients who initially present with early-stage disease will subsequently develop metastases over the course of their disease [2]. Once metastatic CRC (mCRC) is diagnosed, the overall prognosis remains poor with 5-year survival rates in the 8-10% range [3]. Understanding the signaling pathways that regulate the metastatic phenotype will provide the scientific rationale to develop novel agents and/or drug combinations for the treatment of mCRC.

The biological events that drive the initiation, promotion, and progression of CRC occur on several different levels. For example, genetic and epigenetic changes, abnormal activation of EGFR signaling and tumor angiogenesis mediated by VEGF contribute to the progression of mCRC [4]. Several oncogenes have been shown to play important roles in human CRC, such as KRAS, BRAF, and PI3KCA. Oncogenic mutations of KRAS and BRAF occur in approximately 35-40% and 10% of CRC, respectively [5]. Mutations in these proteins result in the constitutive activation of the mitogen-activated protein kinase (MAPK) pathway. The PI3KCA gene encodes the catalytic subunit of phosphatidylinositol 3-kinase (PI3K), and an activating mutation is present in about 20-30% of CRC [6]. PI3KCA mutations with subsequent activation of the AKT pathway have been implicated in the process of colorectal carcinogenesis. In addition, activation of EGFR-mediated signaling results in downstream activation of both MAPK and AKT pathways [7]. Tumor angiogenesis has also been identified as a critical determinant of outcome in mCRC. Patient tumors with high vascular density are more likely to have recurrence and metastasis [8-10]. For this reason, the VEGF-mediated signaling pathway has served as an attractive target for drug development, and two biologic agents, bevacizumab and ziv-aflibercept, are presently approved by the U.S. Food and Drug Administration (FDA) for the treatment of mCRC.

Regorafenib is the first small molecule inhibitor approved by the FDA for the treatment of patients with refractory mCRC who have progressed after standard therapies. It is a multi-kinase inhibitor targeting BRAF, VEGFR-1, -2, -3, KIT, TIE-2, PDGFR-β, FGFR-1, RET, RAF-1, and p38 MAP kinase [11]. However, recent studies have shown that RAF may be the critical target of regorafenib in HCT116 colon cancer cells [12]. In a pivotal randomized phase III study, a statistically significant prolongation in overall survival (OS) was observed in patients with mCRC who received regorafenib therapy. The median OS was 6.4 months in the regorafenib group compared to 5.0 months in the placebo group. These studies also demonstrated a significant improvement in progression-free survival (PFS). The median PFS of the regorafenib-treated group and the placebo group was 2.0 and 1.7 months, respectively [13]. Despite the survival benefit of regorafenib for mCRC patients, its overall clinical efficacy remains quite limited. There is clearly an urgent need to develop more effective approaches for the treatment of mCRC. Rational combination chemotherapy is considered a potentially promising strategy to enhance antitumor activity, overcome cancer drug resistance, and prolong disease-free and overall survival.

Protein kinase D (PKD) is a member of the serine/threonine kinases of the calcium/calmodulin-dependent kinase superfamily, and three main isoforms have been identified: PKD1, PKD2, and PKD3 [14]. This signaling pathway plays a critical role in regulating several important cellular processes, including cell proliferation, survival, angiogenesis, DNA synthesis, adhesion, invasion/migration, and motility [14, 15]. It functions downstream of protein kinase C (PKC), G protein-coupled receptors, and tyrosine kinase receptors. PKD can be activated by both PKC-dependent and independent pathways. In turn, activated PKD phosphorylates a variety of downstream targets at specific sites, such as c-Jun and HDAC IIa, thereby regulating their activity and/or subcellular localization [16, 17]. PKD signaling has been implicated in a wide range of human tumors, including breast, pancreatic, prostate, and glioblastoma. Our laboratory has recently demonstrated that PKD2-mediated signaling pathways, including RAS/RAF/ERK, PI3K/AKT/mTOR, and NF-κB, are activated in human CRC [18]. We also observed that PKD1 was only expressed in normal colon cells and not in human colorectal cancer cells. In contrast, PKD2 and PKD3 were expressed, at the protein and mRNA levels, in both normal and transformed colon cell lines. Given the documented importance of PKD in tumor biology, several PKD inhibitors have been developed, including CRT0066101, CID755673, and kb-NB142-70 [19, 26]. CRT0066101 is a small molecule PKD-specific inhibitor with significant *in vitro* and *in vivo* antitumor activity in human CRC. This molecule inhibited PKD2 activation, blocked NF-κB mediated cellular proliferation and survival, and induced apoptosis [18]. Given the promising therapeutic effect of PKD inhibitors, it is conceivable that the combination of regorafenib with a PKD inhibitor may result in synergistic inhibition of cellular signaling pathways in mCRC. With this in mind, we evaluated the combination of regorafenib and PKD inhibitors using a series of human CRC cell lines, and investigated the downstream signaling effects mediated by this combination were investigated.

## RESULTS

### Effect of the combination of regorafenib and CRT0066101 on CRC cell growth

We evaluated the effect of regorafenib in combination with the pan-PKD inhibitor CRT0066101 on the growth of various human CRC cell lines (HCT116 p53^+/+^, HCT116 p53^−/−^, RKO, HT-29, SW48 and SW48-TP53 [R273H]). As outlined in Table 1, each of these cell lines expressed different gene mutation profiles in the respective KRAS, BRAF, PI3KCA, and TP53 genes. The regorafenib concentration that inhibited 50% of cell proliferation (IC_50_) in these cell lines ranged from 3-6 μM (Table 2). Of note, regorafenib effectively inhibited the growth of TP53 knockout cells (HCT116 p53^−/−^), which has been generally viewed as a CRC cell line resistant to chemotherapy, suggesting that this agent exerts its growth inhibitory effects on CRC growth in a p53-independent manner. Cells with a mutant p53 (HT29) displayed a 2-fold higher IC_50_ value (p<0.05) suggesting that the absence of p53 may be functionally different than having mutant p53 as reported previously [20]. To determine whether the activating p53 mutation (R273H) was the cause of regorafenib resistance, we evaluated the effect of regorafenib on the growth of SW48-TP53(R273H) and its corresponding parental cells. As seen in Table 2, the IC_50_ values of regorafenib in these cell lines were similar suggesting that the p53 activating mutation was not a determinant of regorafenib sensitivity. CRT0066101 was selected for study as previous work had shown that it led to a dose-dependent increase in expression of cleaved PARP and activated caspase-3, in addition to inhibition of AKT and ERK signaling and suppression of NF-κB activity [18]. Moreover, this compound displayed potent growth inhibitory effects against this same panel of human CRC cell lines.

**Table 1:** Mutational gene profile of human CRC cells

Cell Line	KRAS	BRAF	PI3KCA	TP53
HCT116(p53^+/+^)	G13D	WT	H1047R	WT
HCT116(p53^−/−^)	GI3D	WT	H1047R	KO
RKO	WT	V600E	H1047R	WT
HT29	WT	V600E	P449T	R273H
SW48	WT	WT	WT	WT
SW48−	WT	WT	WT	R273H
TP53(R273 H/+)				

The gene mutation status was obtained from the COSMIC website (http://cancer.sanger.ac.Uk/cancergenome/projects/cosmic/).

WT: wild type; KO: gene knockout.

**Fig.1:**
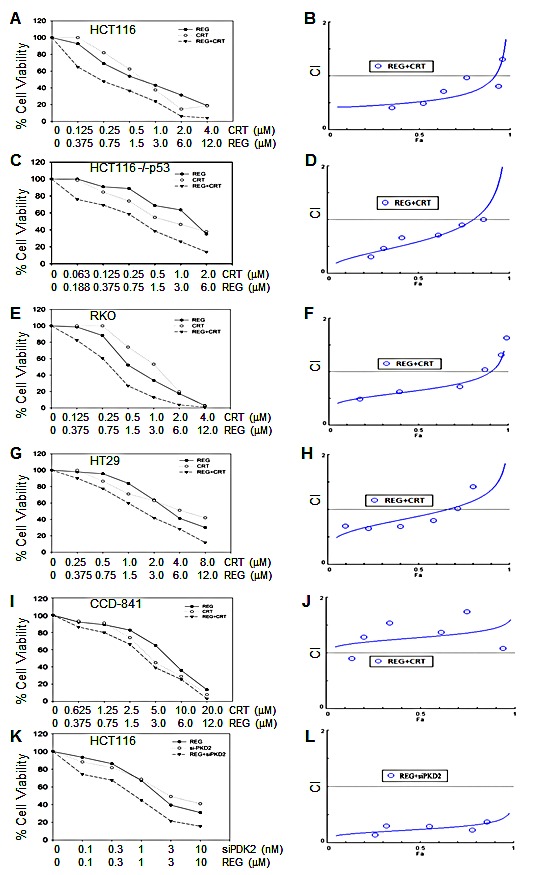
Effect of regorafenib in combination with CRT0066101 on cell growth Human CRC cells (HCT116 (A,B), HCT116 p53^−/−^ (C,D), RKO (E,F), and HT-29 (G,H)) and normal colon CCD-841 epithelial cells (I,J) were seeded in 96-well plates and then treated with different concentrations of regorafenib and/or PKD inhibitor (CRT0066101) for 3 or 7 days (CCD-841 cells). Cell growth was determined by the WST-1 assay as outlined in the Methods section. (K,L) HCT116 cells were treated with PKD2 siRNA complexed with Lipofectamine2000. After 24 hours, culture medium was changed and various concentrations of regorafenib were added. After 72 hours, cell growth was determined by the WST-1 assay as outlined in the Methods section. Data from 3-5 independent experiments were used to calculate the Combination Index (CI) according to the method of Chou and Talalay.

To determine whether simultaneous inhibition of multiple kinases might result in synergistic effects, the combination index (CI) values were calculated according to the Chou-Talalay median effects analysis for drug interactions. Human CRC cells were incubated with various concentrations of regorafenib and PKD inhibitor and at consistent drug ratios for 72 hours. Cell proliferation was determined by WST-1 assay, and the CI values were subsequently calculated for drug interactions using the Calcusyn software [21]. A CI of less than 1.0 was considered to be indicative of synergism, and this interaction was further classified as strong synergism (CI < 0.3), synergism (CI of 0.3-0.7), and slight to moderate synergism (CI of 0.7-0.9). As seen in Fig. 1A, the combination of regorafenib with CRT0066101 exhibited significant synergistic inhibitory effects on the growth of HCT116 cells. The CI value was <1 for concentrations below the respective IC_50_ values for each drug (Fig. 1B). As the drug concentrations reached their IC_50_ values, the effect on cell growth was additive with a CI ~1. To validate that the synergistic effect was due to PKD2 inhibition, we performed growth experiments combining PKD2 siRNA and regorafenib in HCT116 cells. As shown in Fig. 1K, knockdown of PKD2 with a specific siRNA resulted in enhanced cytotoxicity when combined with regorafenib. In contrast, a control siRNA was unable to enhance sensitivity to regorafenib (data not shown). The combination of CRT0066101 and regorafenib demonstrated similar synergistic effects in a number of additional cell lines (HCT116 p53^−/−^, Fig. 1C; RKO, Fig. 1E; HT-29, Fig. 1G). We also evaluated the effects of this combination on the growth of normal colon epithelial CCD 841 CoN cells. In contrast to human CRC cells, the combination treatment did not exhibit synergy in normal colon epithelial cells (Fig. 1I) despite the fact that the individual drug IC_50_ values were similar (Table 2). This finding suggests that the combination of regorafenib and CRT0066101 may preferentially affect CRC cells and spare normal cells from increased toxicity.

**Table 2:** Effect of regorafenib and CRT0066101 on human CRC growth

Cell Line	regorafenib	CRTOO661O1
HCT116(p53^+/+^)	2.90 ± 0.45	0.98 ± 0.13
HCT116 (p53^−/−^)	3.17 ± 0.51	1.01 ± 0.06 *
RKO	3.07 ± 0.06	1.05 ± 029
HT29	5.89±0.88	4.12±0.57
SW48	4.98 ± 0.45	3.42 ± 0.39
SW48-TP53(R273H/+)	5.01 ± 0.32	3.37 ± 0.35
CCD-841	3.36 ± 0.27	5.02 ± 0.16

The cytotoxic effects of regorafenib and CRTOO66IOI on CRC growth were determined by the WST-I assay. IC_50_ (μM) was calculated by Prisrn5.0 software. Values represent the mean ± SD of3 independent experiments performed in duplicate.

* previously published [18].

**Fig.2:**
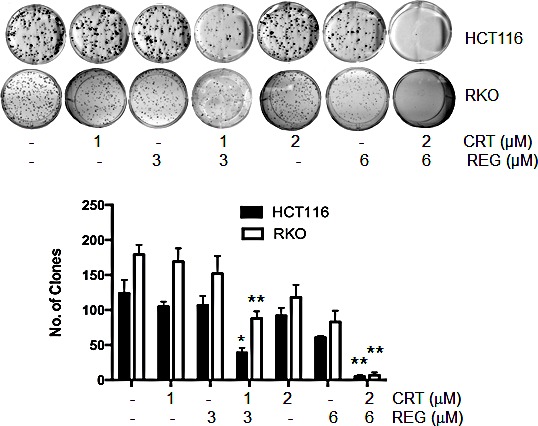
Effect of regorafenib and CRT0066101 on clonogenic growth HCT116 and RKO cells were incubated with different concentrations of regorafenib and/or CRT0066101 for 24 hours. After 10-14 days incubation, clones (>50 cells) were fixed and manually counted. A representative of 4 experiments is shown. *P<0.05, **P<0.01 refers to drug combination group *vs.* corresponding single drug group in HCT116 or RKO cells.

In addition to investigating the effect of the drug combination on cell growth, we determined the potential effect of this combination on clonogenic growth. For these studies, we used HCT116 and RKO cells, and both cell lines were treated with various concentrations of both inhibitors for 24 hours. After an additional 14 days, cell clones were fixed, stained, and counted. As seen in Fig. 2, the combination of regorafenib and CRT0066101 resulted in significant inhibition of clonogenic growth in both the HCT116 and RKO cell lines.

### Effect of the combination of regorafenib and CRT0066101 on apoptosis

**Fig.3:**
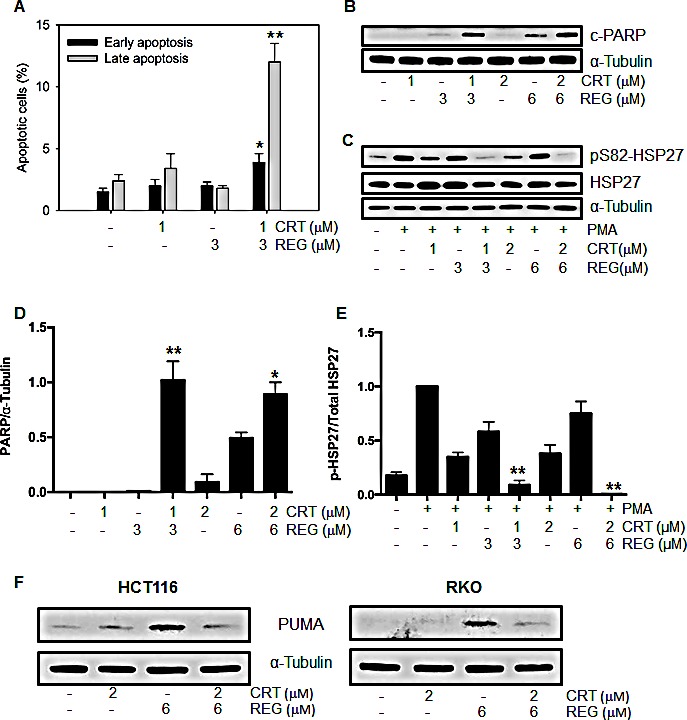
Effect of regorafenib and CRT0066101 on apoptosis RKO cells were exposed to different concentrations of regorafenib and/or CRT0066101 for 24 hours. Cells were processed for FCM to detect apoptotic cells (A) and Western blot analysis for detection of cleaved PARP (B), pSer82-HSP27 and total HSP27 (C) and PUMA (F). Representative blots are shown. (D, E) Values represent the mean ± SD from 3-5 experiments. *P<0.05, **P<0.01 refers to drug combination group *vs.* corresponding single drug group in RKO cells.

Given the synergistic growth inhibitory effect of regorafenib and CRT0066101, we next investigated the potential molecular mechanisms underlying this synergism. To determine the impact of the combination on induction of apoptosis, cells were treated with regorafenib and/or CRT0066101 for 24 hours, stained with FITC-Annexin V and PI, and then analyzed by flow cytometry. As seen in Fig. 3A, treatment with regorafenib or CRT0066101, alone, at their respective IC_50_ concentration, resulted in only minimal induction of apoptosis in RKO cells. However, when the two agents were combined, a significant increase in the number of both early and late apoptotic cells was observed. To explore additional markers of apoptosis, we determined the expression of cleaved PARP and pS82-HSP27. While treatment with regorafenib (3 μM) or CRT0066101 (1 μM), by themselves, had minimal effect on induction of cleaved PARP expression, the combination significantly increased PARP cleavage in RKO cells (Figs. 3B, 3D). In addition, we observed an enhanced reduction in expression of phosphorylated HSP27 with the drug combination. HSP27 has been shown to have strong anti-apoptotic properties through stabilization of the protein translation factor eIF4E in resistant prostate cancer cells [22]. Furthermore, HSP27 is a known substrate of protein kinase D, and abnormal activation of HSP27 may contribute to resistance to apoptosis in human CRC cells [23]. As seen in Figs. 3C and 3E, the combination of regorafenib and CRT0066101 resulted in significant dose-dependent suppression of HSP27 phosphorylation. Similar effects of this drug combination on induction of apoptosis were also observed in HCT116 cells (data not shown).

To further explore the underlying mechanisms mediating the induction of apoptosis, we determined the effect of this drug combination on expression of p53-upregulated modulator of apoptosis (PUMA). PUMA is a member of the Bcl-2 family and has been shown to be an important regulator of apoptosis in CRC. Recent studies have demonstrated that regorafenib induces PUMA expression in a time- and concentration-dependent manner [12] but it is not yet known whether PKD inhibition directly activates this pathway. As seen in Fig. 3F, CRT0066101 treatment had no effect on PUMA expression while regorafenib induced its expression as expected. Interestingly, the combination of regorafenib and PKD inhibition resulted in suppression of PUMA induction. Thus, our results suggest that the combination of regorafenib with CRT006601 significantly induced apoptosis in human CRC.

### Effect of the combination of regorafenib and CRT0066101 on activation of ERK and AKT signaling

**Fig.4:**
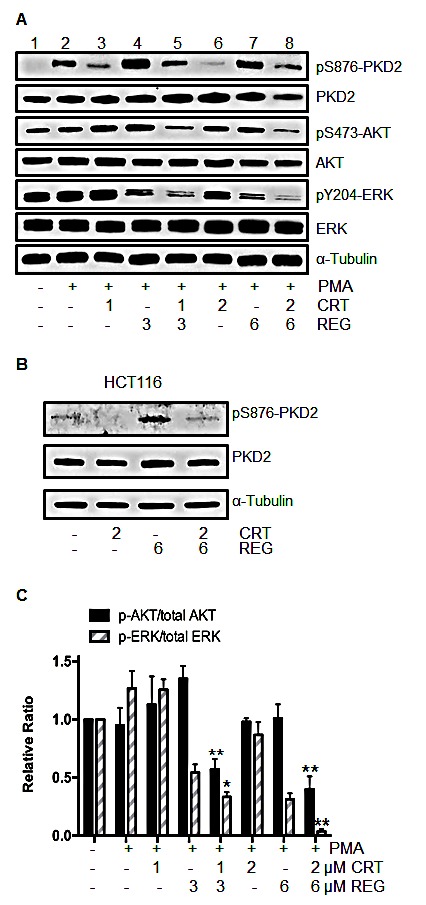
Effect of regorafenib and/or PKD inhibitor CRT0066101 on activation of ERK and AKT signaling (A) RKO cells were exposed to regorafenib and/or CRT0066101 for 24 hours, stimulated with 100 nM PMA for 30 minutes, then processed for Western blot analysis of pSer876-PKD2, pS473-AKT, pY204-ERK, total PKD2, AKT and ERK. A representative experiment is shown. (B) HCT116 cells were exposed to regorafenib and CRT0066101 for 24 hours and the level of pSer876-PKD2 and PKD2 protein expression was determined. (C) Values represent the mean relative ratios ± SD from 3-5 experiments. *P<0.05, **P<0.01 refers to drug combination group *vs.* corresponding single drug group of RKO cells.

The RAS/RAF/ERK, PI3K/AKT/mTOR, and NF-κB signaling pathways are well-established survival pathways in human CRC. Constitutive activation of each of these pathways has been shown to activate cell proliferation and the development of cellular drug resistance [5, 24]. Our earlier studies had shown that PKD2 plays an important role in mediating growth and survival signaling pathways in CRC [18]. Similarly, the multi-kinase inhibitor regorafenib is able to simultaneously suppress RAS/RAF/ERK and PI3K/AKT/mTOR pathways [25]. With this in mind, we investigated the potential effect of the drug combination on activation of these key signaling pathways. After exposure to regorafenib and/or CRT0066101 for 24 hours, p-PKD2, p-ERK, p-AKT and total PKD2, ERK and AKT expression was determined by Western blot analysis. As anticipated, CRT0066101 effectively blocked the activation of PKD2 (Fig. 4A, lanes 3 and 6). Treatment of regorafenib alone demonstrated no suppressive effect on PKD2 activity, and in fact, appeared to result in increased PKD2 activation. To investigate this further, HCT116 cells were treated with the drug combination without PMA stimulation. As seen in Fig. 4B, treatment with regorafenib alone resulted in induction of p-PKD2 expression. The combination of the two agents prevented this induction. As p-PKD2 was undetectable in non-stimulated RKO cells, no induction was observed in these cells (data not shown). With respect to the downstream pathways, we observed that regorafenib or CRT0066101, each by itself, did not affect expression of p-AKT. However, the two drugs in combination caused a significant reduction in p-AKT expression without altering total AKT levels (Fig. 4A, lanes 5 and 8). In addition, treatment with 1 μM of CRT0066101 had little effect on p-ERK expression (Fig. 4A, lane 3). However, the combination of CRT0066101 (1 μM) and regorafenib (3 μM) yielded a significantly reduced expression of p-ERK (Fig. 4A, lane 5) while the expression of total ERK was not altered. The higher dose combination resulted in a greater reduction in expression of p-ERK in RKO cells (Fig. 4A, lanes 5 and 8). Similar effects of this drug combination were also observed in HCT116 cells. Taken together, these findings suggest that PKD2 inhibition of may lead to an enhanced inhibitory effect of regorafenib on both ERK and AKT signaling.

### Effect of regorafenib and CRT0066101 on NF-ΚB activity

**Fig.5:**
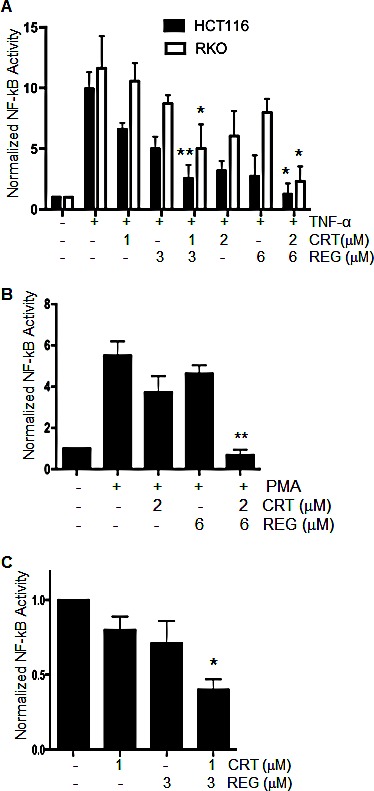
Effect of regorafenib with CRT0066101 on NF-κB activity (A) HCT116 and RKO cells were transiently transfected with a luciferase plasmid under the control of a NF-κB response element. On the following day, cells were treated with regorafenib and/or CRT0066101 for 2 hours, and then stimulated with TNF-α for an additional 5 hours. NF-κB activity was determined by the dual-luciferase assay. *P<0.05, **P<0.01 means drug combination group *vs.* corresponding single drug group in HCT116 or RKO cells. (B) RKO cells stably expressing NF-κB-luciferase were treated with regorafenib and/or CRT0066101 for 2 hours, and then stimulated with PMA for an additional 5 hours. NF-κB activity was determined by the dual-luciferase assay. **P<0.01, refers to drug combination group *vs.* corresponding single drug group in RKO cells. (C) RKO cells stably expressing NF-κB-luciferase were treated with regorafenib and/or CRT0066101 for 24 hours, and NF-κB activity was determined. Luciferase values represent the mean ± S.D. from 3-5 separate experiments. *P<0.05, *vs.* untreated cells.

Previous studies from our lab and others have shown that CRT0066101 treatment has a suppressive effect on activation of NF-κB [18, 26]. As regorafenib is a multi-kinase inhibitor, it is conceivable that it may also impact on NF-κB signaling. For this reason, the combination of regorafenib and CRT0066101 may result in a synergistic inhibitory effect on NF-κB activation. To investigate the effect of the drug combination on NF-κB activity, HCT116 and RKO cells were each transiently transfected with a luciferase plasmid under the control of the NF-κB response element. After incubation of regorafenib and/or CRT0066101 for 2 hours, cells were stimulated by TNF-α (50 ng/mL) for an additional 5 hours, and NF-κB activity was determined by the dual luciferase assay. As seen in Fig. 5A, both regorafenib or CRT0066101, as single agents, inhibited NF-κB activation, while the combination of regorafenib and CRT0066101 significantly enhanced the inhibitory effect of NF-κB activity in a dose-dependent manner. In addition to TNF-α stimulation, we stimulated NF-κB activity with PMA, a known PKD pathway activator. While each drug alone had minimal effect, the combination of the two molecules significantly inhibited PMA-induced NF-κB activity (Fig. 5B). We also determined the effect of this combination on basal levels of NF-κB activity. The effect of combined regorafenib and CRT0066101 enhanced the suppression of basal NF-κB activity (Fig. 5C), which is a finding consistent with the TNF-α- and PMA-induced results. Taken together, the combination of regorafenib with CRT0066101 resulted in a synergistic suppression of activation of NF-κB pathway.

## DISCUSSION

The direct targeting of multiple cell signaling pathways such as EGFR and VEGF has proven to be a promising strategy to treat patients with mCRC [27, 28]. However, only a subgroup of patients with this disease who are wild-type RAS and BRAF will be responsive to anti-EGFR therapy. Similarly, the clinical benefit of targeted VEGF approaches in prolonging overall survival of mCRC patient is limited, and a fraction of patients are intrinsically refractory or acquire resistance [28-30]. As a result, there is a significant unmet medical need for the development of effective therapies that can stabilize and/or slow the progression of mCRC. Thus, the development of novel drug combination regimens is considered a potentially effective strategy to enhance antitumor activity, overcome cellular drug resistance, and prolong PFS and OS [31]. With this in mind, we used the Chou-Talalay median effects analysis to demonstrate that the combination of regorafenib with a PKD inhibitor resulted in a significant synergistic cytotoxic effect in CRC cells that expressed different genetic mutation profiles.

Various DNA mutation profiles in genes such as KRAS, BRAF, PI3KCA, and TP53 have been associated with the efficacy of targeted therapy, cellular drug resistance, and poor overall prognosis. KRAS, BRAF, or PIK3CA mutation results in a continuous activation of the downstream RAS/RAF/ERK or PI3K/AKT/mTOR pathways, regardless of whether the EGFR is activated or blocked [5]. Such activation in turn enhances transcription of various oncogenes, including MYC, CREB, and NF-κB [32]. The presence of KRAS mutations, which occur in 35-40% of CRC patients, has emerged as a key determinant of resistance to targeted EGFR treatment. TP53, one of the most frequently mutated genes in CRC, is a critical regulator gene controlling several important cellular processes, such as cell cycle, apoptosis, DNA repair, and genomic integrity [33]. Clinical investigators have demonstrated that there is an association between somatic mutations in TP53 or abnormal protein expression with poor survival or lack of response to therapy [34].

In the present study, we evaluated the combined effect of multi-kinase inhibitor regorafenib and pan-PKD inhibitor CRT0066101 against 4 human CRC cells (including HCT116, HCT116 p53^−/−^, RKO and, HT-29), with each cell line having different mutation profiles in KRAS, BRAF, PI3KCA, and TP53 genes. Our data show that the combination of regorafenib with CRT0066101 displayed synergistic effects in CRC cells that expressed mutations in these important genes. Interestingly, this combination was not synergistic in normal colon epithelial cells, which suggests that this combination might exhibit an improved selective advantage in tumor cells over normal, thereby resulting in a potential improved therapeutic window. We further demonstrated that this drug combination activated the apoptotic pathways, perhaps mediated through a PUMA-independent manner as PUMA induction normally observed with regorafenib treatment was suppressed in the presence of both drugs.

As regorafenib inhibits multiple kinases and PKD regulation can alter cellular survival signaling cascades, we determined the effect of drug combination on these critical pathways. Our results show that regorafenib combined with CRT0066101 synergistically suppressed RAS/RAF/ERK and PI3K/AKT/mTOR pathways in CRC (Fig. 4). Activation of NF-κB signaling is often observed in CRC, and this pathway plays a critical role in facilitating oncogenesis and metastasis and in mediating resistance to chemotherapy and radiation therapy [24]. PKD is a key mediator of NF-κB activation in pancreatic cancer and human CRC. In pancreatic cancer, the PKD inhibitor CRT0066101 significantly suppressed NF-κB activity and the expression of NF-κB–dependent gene products essential for cell proliferation and survival [26]. Similarly, our previous studies showed that structurally distinct PKD inhibitors (CRT0066101 and kb-NB-142-70) all substantially suppressed the activation of NF-κB in human CRC [18]. In the current study, we showed that the combination of regorafenib with CRT0066101 synergistically suppressed the activation of NF-κB pathway in both stimulated CRC cells and under basal conditions (Fig. 5). This observation may explain the lack of PUMA induction seen with this drug combination as PUMA expression is regulated by NF-κB [12]. Taken together, this combination effectively blocked cancer cellular survival signaling cascades. Of note, regorafenib, as a single agent, was able to induce the phosphorylation of PKD2. This activation may represent a cellular resistance pathway by which the cells maintain growth and proliferation in the presence of exposure to the multi-kinase inhibitor. However, when combined with the PKD inhibitor, this activation was effectively suppressed providing additional evidence supporting this drug combination.

**Fig.6:**
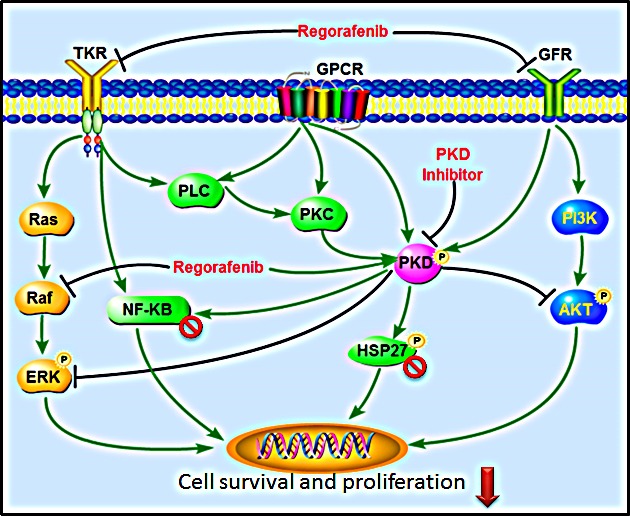
Signaling pathways altered by the combination of regorafenib and PKD inhibitors The combination of regorafenib and PKD inhibitor leads to a synergistic inhibition of survival signaling cascades, including RAS/RAF/ERK, PI3K/AKT/mTOR, and NF-κB pathways.

In conclusion, we have shown that targeted inhibition of PKD is able to significantly enhance the cytotoxic effect of regorafenib in a series of human CRC cell lines. The mechanisms underlying this positive drug-drug interaction may be associated with synergistically induced apoptosis and inhibition of key survival signaling cascades, including RAS/RAF/ERK, PI3K/AKT/mTOR and NF-κB pathways (Fig. 6). These findings suggest that the combination of regorafenib and a PKD inhibitor may represent a novel strategy for the treatment of mCRC.

## MATERIALS AND METHODS

### Materials

Regorafenib was purchased from Selleck Chemicals LLC. CRT0066101 was kindly provided by Cancer Research Technology Inc. This agent is a slight yellow powder with 99.0% purity, and freshly dissolved in dimethyl sulfoxide before use (final concentration of DMSO in cell culture medium is less than 0.1%). WST-1 was purchased from Roche (Indianapolis, IN). DMSO and all other chemicals were obtained from Sigma. PKD2-specific siRNA (sense 5′-UGAGACACCUUCACUUCAA-3′; #D-004197-05) and control siRNA (sense 5′-GGAUACUGCCAAUCUCUAGG-3′) were purchased from Thermo Fisher Scientific Inc. (Waltham, MA).

### Cell lines and cell culture

HCT116 p53^+/+^ and p53^−/−^cell lines were kindly provided by Dr. Bert Vogelstein. SW48 and SW48TP53 (R273H/+) cell lines were purchased from Horizon Discovery Ltd. (Cambridge, United Kingdom). The human colon cancer RKO and HT-29 cell lines and the normal colon epithelial CCD-841 cell line were obtained from ATCC. All cell lines were maintained in RPMI-1640 (Invitrogen; Carlsbad, CA) with 10% (v/v) fetal bovine serum at 37°C in a humidified incubator with 5% CO_2_. HCT116 and RKO cells were profiled at the University of Pittsburgh Cell Culture and Cytogenetics Facility (August 2013 authenticated by STR). Cells were tested monthly for mycoplasma by the MycoAlert Mycoplasma detection assay (Cambrex BioScience).

### Cell proliferation and clonogenic assays

Human CRC cells were seeded in 96-well plates at 1500-2000 cells/well. On the following day, cells were treated with various concentrations of regorafenib and/or CRT0066101 for 72 hours. For siRNA/regorafenib combination experiments, siRNAs were transfected using Lipofectamine 2000 at various concentrations. After 24 hours, the medium was replaced and regorafenib was added for an additional 72 hours. Cell viability was determined by the WST-1 assay (Roche; Indianapolis, IN). The IC_50_ value was defined as the drug concentration that inhibits 50% cell growth compared with untreated controls and calculated by Graphpad Prism 6.0 software. To determine the potential mechanism of drug-drug interactions, we used CalcuSyn software (version 2.0, Biosoft, Cambridge, UK) to calculate the combination index (CI) according to published methods. A CI of less than 1.0 was considered to be indicative of synergism, and this interaction was further classified as strong synergism (CI < 0.3), synergism (CI of 0.3-0.7), and slight to moderate synergism (CI of 0.7-0.9).

For clonogenic assays, HCT116 and RKO cells were seeded in 6-well plates at a density of 400 cells/well. On the following day, cells were treated with various concentrations of regorafenib and/or CRT0066101 for 24 hours, after which time, the growth medium was replaced. After 10-14 days, clones were fixed with trypan blue solution (75% methanol/25% acetic acid/0.25% trypan blue) for 15 minutes, washed, and air-dried before counting clones >50 cells.

### Western blot analysis

Protein concentration of cell lysates was determined by the DC Protein Assay (Bio-Rad; Hercules, CA). Equal amounts of protein (30 μg) from each cell lysate were resolved on SDS-PAGE using the method of Laemmli and transferred onto 0.45 μm nitrocellulose membranes (Bio-Rad). Membranes were blocked and incubated overnight with primary antibodies at 4°C. The following antibodies were used in the experiments: anti-pTyr204-ERK (#sc-7383; Santa Cruz Biotechnology), anti-ERK (#sc-94; Santa Cruz Biotechnology), anti-pSer473-AKT (#9542; Cell Signaling), anti-AKT (#9272; Cell Signaling), anti-pSer867-PKD2 (#07-385; Upstate), anti-PKD2 (#07-488, Upstate), anti-PARP (#9542; Cell Signaling), anti-p-Ser82-HSP27 (#9709; Cell Signaling), anti-HSP27 (#2402; Cell Signaling), anti-PUMA (#12450; Cell Signaling) and anti-α-tubulin (EMD Biosciences; San Diego, CA). After multiple TBST washes (1×TBS, 0.1%Tween-20), membranes were incubated with corresponding horseradish peroxidase-conjugated secondary antibodies (Bio-Rad) for 1 hour at room temperature. Proteins were detected by the enhanced chemiluminescence method (SuperSignal West Pico substrate; Pierce; Rockford, IL). Quantitation of signal intensities was performed by densitometry on a Xerox scanner using ImageJ software.

### Apoptosis assay

Cells were seeded in 6-well plates at a density of 4×10^5^ cells/well. After exposure to different concentrations of regorafenib and/or CRT0066101 for 24 hours, cells were harvested, washed twice with PBS, resuspended with 1x binding buffer (BD Biosciences; San Jose, CA), stained with FITC Annexin V Apoptosis Detection Kit (BD Biosciences; San Jose, CA), and analyzed on the BD Accuri C6 Flow Cytometer (BD Accuri Cytometers Inc.; Ann Arbor, MI) at the UPCI Flow Cytometry Facility.

### NF-κB activity assay

Cells (1×10^5^) were plated in 24-well plates 16 hours before transfection. A total of 0.5 μg of pGL3-Luc-NF-κB DNA was then co-transfected with 0.1 μg of Renilla luciferase vector DNA (internal control, Promega) into cells using Lipofectamine 2000 according to the manufacturer's instruction (Invitrogen). After 6 hours, the growth medium was changed. On the following day, cells were treated with various concentrations of regorafenib and/or CRT0066101 for 2 hours, followed by stimulation with TNF-α (50 ng/mL) or PMA (100 nM) for an additional 5 hours. Luminescence values were normalized with renilla activity, and the reporter assays were performed in triplicate. For measurement of basal NF-κB activity, RKO cells stably expressing the pGL3-Luc-NF-κB construct were used [17]. Luminescence values were normalized to total soluble protein as measured by the Pierce 660 nm Protein Assay (Thermo Scientific, Inc.).

### Statistical analysis

Data were expressed as mean ± SD. Statistical analysis of the data was performed using SPSS software the one-way ANOVA. P<*0.05* was considered statistically significant.
